# The legacies of the “Father of Hybrid Rice” and the seven representative achievements of Chinese rice research: A pioneering perspective towards sustainable development

**DOI:** 10.3389/fpls.2023.1087768

**Published:** 2023-03-21

**Authors:** Jian-Guo Gao, Xin-Guang Zhu

**Affiliations:** ^1^Xinjiang Key Laboratory of Biological Resources and Genetic Engineering, College of Life Science and Technology, Xinjiang University, Urumqi, China; ^2^Department of Ecology, Peking University, Beijing, China; ^3^National Key Laboratory for Plant Molecular Genetics, Center for Excellence in Molecular Plant Sciences, Chinese Academy of Sciences, Shanghai, China

**Keywords:** hybrid rice, sea rice, *de novo* domestication, perennial rice, food security, sustainable development goals

## Abstract

The “Father of Hybrid Rice”, Yuan Longping, created high-yield hybrid rice that can feed tens of millions of people annually. The research achievements of Yuan and his team on low cadmium-accumulating rice and sea rice, in addition to hybrid rice, as well as those of a large number of Chinese scientists engaged in rice research in other six areas, including the rice genome, purple endosperm rice, *de novo* domestication of tetraploid rice, perennial rice, rice blast disease, and key genes for high nitrogen use efficiency, play an important role in promoting the realization of the United Nations Sustainable Development Goals 2 and 12. The purpose of this review is not to elaborate on the details of each research, but to innovatively summarize the significance and inspiration of these achievements to ensure global food security and achieve sustainable agriculture. In the future, cultivating new rice varieties through modern biotechnology, such as genome editing, will not only reduce hunger, but potentially reduce human-land conflicts, improve the environment, and mitigate climate change.

## 1 Introduction

Yuan Longping (1930–2021), respectfully named by an Indian scholar as the “Father of Hybrid Rice” in 1982, continues to make important contributions to the research, promotion, and commercialization of hybrid rice (Yao, 2015). Through the establishment of close collaborations between farmers and the Chinese government, Yuan and his colleagues promoted large-scale hybrid rice cultivation in China in 1976. Over the past half century, this led to an increase in the rice cultivation are to 8 billion mu (1 mu = 0.067 hectares) and cumulatively increased the output of rice to more than 600 billion kg, helping to ensure national food safety. At the same time, hybrid rice has also been successfully promoted in other countries and regions in the world, with 78 million mu devoted to hybrid rice cultivation in more than 70 countries and regions in Southeast Asia, South Asia, South America, and North America. Therefore, hybrid rice has been listed by the Food and Agriculture Organization (FAO) of the United Nations as the first technology for these countries to help solve food shortages (Zhu, 2016).

## 2 Core scientific ideas of hybrid rice and rice yield

Born in poor and backward China (before the founding of the People’s Republic of China), Yuan witnessed the tragic images of starvation as a youth (Wu, 2021), which prompted him to pursue research on rice breeding in an attempt to solve the problem of starvation. Yuan’s landmark research began with a field survey in 1964; after inspecting 14,000 rice ears, he found six male sterile individuals, and published this discovery two years later (Yuan, 1966). Inspired by heterosis, or hybrid vigor, found in corn and sorghum, Yuan firmly believed that heterosis would also be identified in rice, even though this idea went against the mainstream academic view at the time. The prevailing view was that “rice is a self-pollinated plant; thus, there will be no heterosis.” Later, Yuan innovatively used the “three-line matching method” (first-generation hybrid rice technology) to verify heterosis in rice, and subsequently developed a two-line method that greatly improved the efficiency of rice breeding and seed production (i.e., a photoperiod/thermosensitive genic male sterile line; second-generation hybrid rice technology) and super hybrid rice (third-generation hybrid rice technology) (Qian et al., 2016; Yuan, 2016). Thanks to Yuan’s genius idea, ​​“combination of morphological improvement and utilization of heterosis”, for super-high-yield hybrid rice breeding in 1996, the Ministry of Agriculture of China formally approved China’s Super Rice Breeding Program, and considerable progress was made. The reported yields of 926.6 kg/mu and 1026.7 kg/mu in 2011 and 2014, respectively, were twice the average yield of rice in China (Wu et al., 2016). In 2020, the production data of third-generation hybrid rice in Hengnan County, Hunan Province, showed that the yield of cross-season rice reached 1530.76 kg/mu, which is a remarkable breakthrough in the history of rice cultivation.

## 3 Two dreams

When Yuan was in his 20s, he had a dream, “enjoy the cool underneath the rice crops” (Yao, 2015). For many years, he and his team were committed to research on high-quality and high-yield rice, which has been successfully planted in other parts of the world. Another bolder dream, “hybrid rice to be grown all over the world”, was aimed at helping to solve global food scarcity (Wang, 2021). Yuan established the Hunan Hybrid Rice Research Center in 1984 to train and educate many rice research scientists. In addition to continuing research on hybrid rice, his team has also conducted fruitful research on low cadmium-accumulating rice and sea rice in recent years. For example, through genome editing, site-specific mutations of key genes involved in cadmium uptake by rice can effectively block the absorption of cadmium, reducing its content in rice (Tang et al., 2017; Wang et al., 2021).

**Figure 1:**
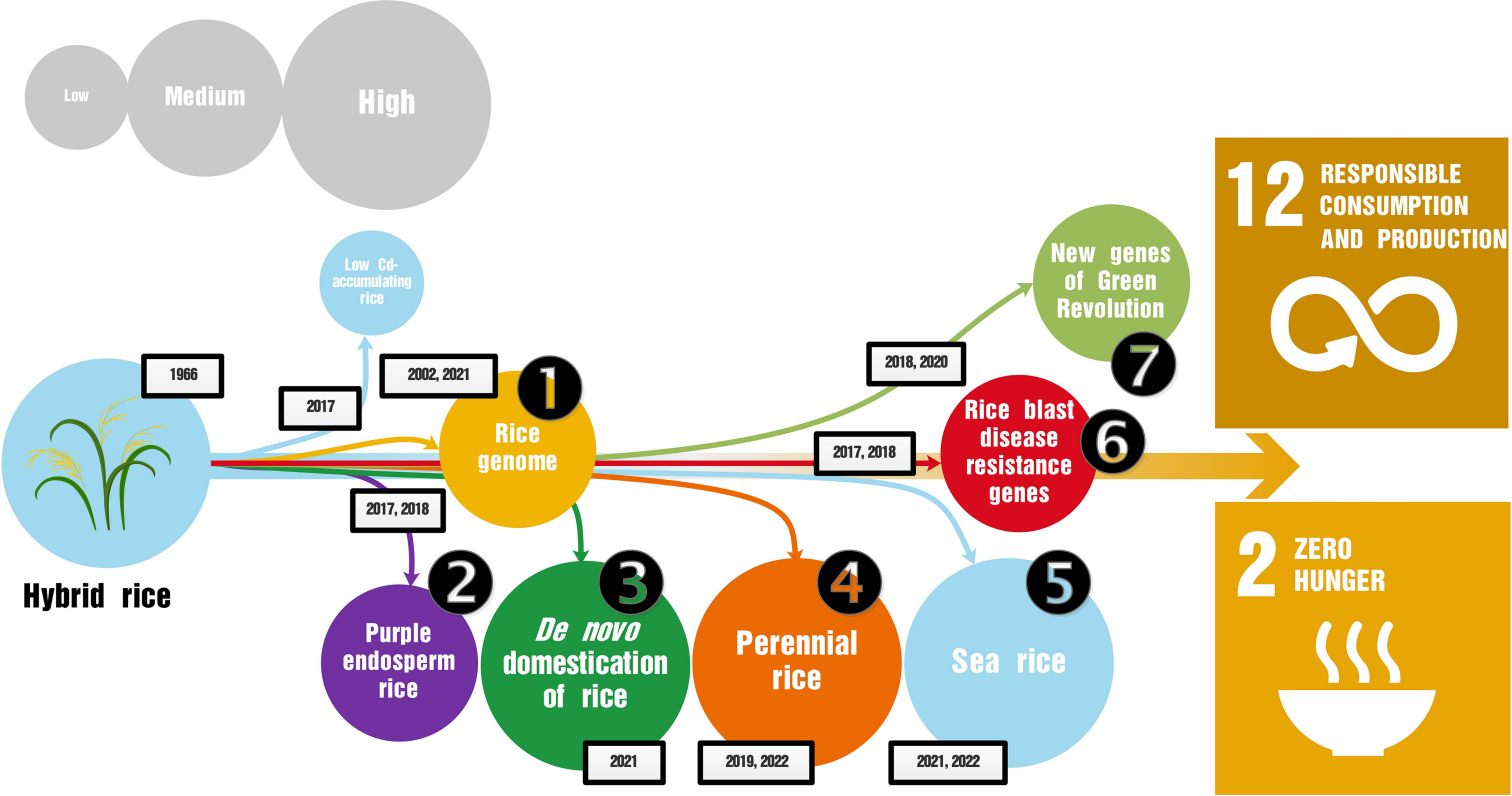
Evolution of the shifting rice research themes that were originally derived from hybrid rice. In addition to hybrid rice, low cadmium-accumulating rice and sea rice were mainly developed by Yuan Longping and his team (indicated by blue circles and lines). The remaining six research areas carried out by Chinese rice scientists are shown in the order discussed in this article (circled hollow numbers); numbers in the boxes outlined in black indicate the breakthrough year for each area of research, but not the year when the research was initiated. The importance of these research studies to the achievement of the Sustainable Development Goals is represented by the size of the circle, which is arbitrary. According to the nature of the research and the inherent relevance to the Sustainable Development Goals, these research areas are divided into different groups; for example, sea rice, *de novo* domestication of rice, perennial rice, and purple endosperm rice are conducive to the realization of Goal 2; low cadmium-accumulating rice and research on new Green Revolution varieties of rice are conducive to the realization of Goal 12; whereas research on the rice genome and rice blast disease promote the realization of both goals simultaneously.

Owing to continuous breakthroughs and great achievements in hybrid rice breeding and promotion, Yuan has long been a symbolic figure of China’s rice research program. He had a significant influence on the R&D policies of the Chinese government and greatly influenced China’s rice research. Today, China has the world’s largest team of scientists studying rice; there are at least 50,000 scientists in 3000 laboratories across the country engaged in rice research, contributing more than half of the world’s publications in this field (Jia, 2018). Below, we have summarized seven areas of study of great significance to world food security that were inspired by Yuan’s success, but some of them were not carried out by Yuan or his team directly. The achievements of these studies are of great significance in reducing malnutrition, reducing the use of chemical fertilizers and pesticides in the process of rice production (**Figure 1**), and in achieving the United Nations Sustainable Development Goals (Chen et al., 2022).

### 3.1 Rice genome

Yuan might have been one of the very early Chinese geneticists who was influenced by modern genetics. He attached great importance to the application of molecular biology to improve the efficiency of rice breeding and participated in the Chinese rice genome sequencing project. As recently as ~20 years ago, Chinese rice scientists discovered that only 466 Mb of the rice genome contained rich genetic variations, with 46,022–55,615 protein-coding genes (Yu et al., 2002). In 2018, a number of institutions from China jointly released the world’s first, nearly complete, and high-quality pan-genome of Asian cultivated rice (Wang et al., 2018b). This research resulted in the renaming of *indica* and *japonica* rice, and provided the most refined cultivated rice populations, which contain a wealth of genomic structural variants (SVs). Recently, based on the genome information of two *indica* rice varieties, ZS97 and MH63, Chinese scientists discovered a large number of SVs at the end of the long arm of chromosome 11, located in the centromeric region, and a large number of resistance genes, which was the first reported gap-free plant reference genome (Song et al., 2021).

### 3.2 Purple endosperm rice

Every year, billions of people suffer from undernourishment worldwide. In 2019, approximately 3 billion people worldwide did not have access to a healthy diet; worse, 22.0% of children under the age of five (149.2 million children) were stunted due to malnutrition, which is particularly pervasive in many developing countries in Africa and Asia (FAO et al., 2021). The Rockefeller Foundation proposed a rice breeding program to increase the beta-carotene content in “Golden Rice” in 1982. However, public misperceptions caused by false non-governmental organization (NGO) narratives have resulted in only a very few countries approving “Golden Rice” for cultivation and commercial production (Qaim, 2020). Chinese geneticist Liu Yaoguang used synthetic biology and a multi-gene stacking system to develop “purple endosperm rice”, which is rich in anthocyanins in the light purple endosperm and has enhanced nutrition (Zhu et al., 2017). Anthocyanins have excellent anti-aging effects and while they are found in many grains, they are naturally only present in the seed coat and not in the endosperm. In addition to developing rice with high anthocyanin content, astaxanthin-containing rice has been produced in the laboratory through genetic engineering and synthetic biology (Zhu et al., 2018). Astaxanthin is an orange-red carotenoid pigment generally produced by some algae and bacteria, but most land plants cannot synthesize it. The pigment is the highest-graded carotenoid and one of the strongest antioxidants discovered to date; astaxanthin exhibits strong free radical scavenging ability, anti-aging properties, and protective effects against cardiovascular disease. Therefore, large-scale promotion of the cultivation of “purple endosperm rice” has great beneficial potential for eliminating malnutrition on a global scale and improving human health.

### 3.3 *De novo* domestication of tetraploid rice

Cultivated rice is diploid, a cultivated species that has been domesticated from its diploid ancestor over thousands of years. However, in addition to diploid cultivated rice, the *Oryza* genus includes 25 other wild plants, which can be classified into 11 categories according to their genome characteristics, including 6 types of diploid genomes and 5 types of tetraploid genomes. Among these, the allotetraploid wild variety combines the two complete diploid chromosomes of the CC and DD genomes, which have natural heterosis and also the advantages of large biomass and strong environmental adaptability. Chinese geneticist Li Jiayang proposed a rapid *de novo* domestication strategy for allotetraploid wild relatives to reduce their domestication time, which typically requires thousands of years. The research team identified a high-quality allotetraploid polyploid rice 1 (PPR1) wild relative that originated from tropical regions of South America with an extremely large biomass; the plant height can reach 2.7 m, the ear length can reach 48 cm, and the leaf width can reach 5 cm. The researchers assembled the first allotetraploid rice reference genome with a size of 894.6 Mb, which is about twice the size of cultivated rice. More than 81,000 high-confidence genes were annotated, including 10 domesticated-related genes and 113 important agronomic trait genes found in cultivated rice. Through gene editing of these genes, rapid domestication has been achieved (Yu et al., 2021). This study is the first to demonstrate *de novo* domestication of a wild crop and indicates that *de novo* domestication may facilitate cultivation of a new staple food crop (Zhu and Zhu, 2021).

### 3.4 Perennial rice

Traditional cultivated rice is an annual crop, which means that seeds need to be sown every year. Scientists from Yunnan University have cultivated a perennial rice, through traditional hybridization of *O. sativa* L. and *O. longistaminata* which was rice variety native to Niger. After one-time planting, a crop can be harvested for the next 3–5 years (Zhang et al., 2019), greatly improving planting efficiency, and saving seed, fertilizer, and labor. Recent data has shown that the average yield of summer rice in July is 655.65 kg/mu, the average yield of autumn rice in October is 464.8 kg kg/mu, and the average yield of perennial rice is 1120.45 kg/mu. Thus, perennial rice has significant potential for large-scale planting. In a study on the combined effects of nitrogen application and planting density for four consecutive harvest seasons, researchers found that perennial rice maintained the best regrowth ability at a higher planting density, and lower level of nitrogen fertilizer. This suggests that perennial rice has the potential to use less chemical fertilizer while still producing high yields, which is conducive to green and sustainable agriculture (Zhang et al., 2021). Importantly, the profit for farmers from planting perennial rice currently averages at about 235% higher than that from planting annual rice (Zhang et al., 2021). In addition, owing to the reduction in plowing of perennial rice fields, soil erosion can be reduced, protecting arable land (Huang et al., 2018), and potentially promoting the accumulation of soil organic carbon, mitigating climate change. The commercialized rice was successfully grown on more than 15,000 ha by ~45,000 smallholder farmers in southern China in 2021, contributing to crop productivity, farmer livelihoods, and soil health (Zhang et al., 2022). By leveraging wild rice relatives, perennial rice’s success is inspiring for other crops’ breeding and social, economic, and environmental sustainability (Crews and Cattani, 2018; DeHaan et al., 2020; Zhang et al., 2022).

### 3.5 Sea rice

The team led by Yuan Longping has a great ambition in promoting sea rice. By leveraging saline-alkali land improvement projects supported by the central government, the team plans to plant sea rice in 100 million mu in 8–10 years. Due to the accelerated biotechnology advancements in rice research (Chen et al., 2022; Shang et al., 2022), there is a great potential for large-scale improvement in its production along the Chinese seashore. Currently, fields in Panjin exhibit an average yield of only 300 kg/mu, half that of traditional rice fields. A breakthrough in sea rice technology could facilitate turning at least 300 million mu of saline-alkali land into grain fields, which is equivalent to 1/6 of China’s arable land. A recent study on rice salt stress showed that the gene *RST1* (*rice salt tolerant 1*), a negative regulator played a crucial role in rice survival under saline conditions (Deng et al., 2022). Malfunction of *RST1* promotes nitrogen utilization and reduces ${\text{NH}}_{4}^{+}$ accumulation subjected to salt stress, and, increases grain yield, which laid a solid foundation in sea-water tolerant rice breeding (Deng et al., 2022).

### 3.6 Rice blast disease

During the entire growth period of rice, the plant is susceptible to infection by various pathogens. Rice blast, also called rice cancer, is caused by the fungus *Magnaporthe oryzae*; it has the greatest impact on yield causing a 10–35% reduction in yield or even no harvest (Li et al., 2019). Identifying resistance genes in rice to *M. oryzae*, especially long-lasting broad-spectrum resistance genes, has been a long and difficult task for rice breeders. Recently, researchers discovered a key role for Ideal Plant Architecture 1 (IPA1), a key transcription factor for the establishment of ideal plant types in rice, in combatting rice blast disease (Wang et al., 2018a). Interestingly, IPA1 promotes growth under normal conditions, and improves the immune response when a plant is infected by the fungus, providing an important theoretical basis for high-yield and high-resistance breeding. Through the combination of big data analysis in genetic, biochemical, pathological, and other experimental methods, researchers have discovered the gene *bsr-d1*, which confers broad-spectrum and long-lasting disease resistance to rice blast caused by *M. oryzae* (Li et al., 2017). The researchers also discovered a recessive gene, *bsr-k1*, which also confers broad-spectrum resistance to rice blast (*M. oryzae*), as well as to bacterial blight, caused by the proteobacteria *Xanthomonas oryzae*. The immune response activated by *bsr-k1* is relatively mild and has no significant effect on the agronomic traits of rice (Zhou et al., 2018). Every year, China loses at least 16 million tons of food due to pathogens and insect pests, which is equivalent to feeding 300 million people annually (Deng et al., 2021). Therefore, the discovery of these transcription factors and disease resistance genes indicates that pathogen resistance of rice may be improved through gene editing in the future (Deng et al., 2017; Gao et al., 2021), which is extremely important for obtaining a stable rice yield.

### 3.7 High nitrogen use efficiency

In the 1960s, Green Revolution, the large-scale promotion of semi-dwarf rice and wheat varieties, effectively solved the problems of plant lodging and yield reduction caused by large-scale fertilization, and greatly increased the output of the world’s major food crops (Evans and Lawson, 2020). Although the semi-dwarf varieties developed as part of the Green Revolution have excellent characteristics of high fertilizer tolerance, lodging resistance, and high yield, they also have the disadvantage of low nitrogen use efficiency, which leads to a high dependence on chemical fertilizers (Li et al., 2018). To increase crop yields, and continuous application of a large amount of nitrogen fertilizer is required, but this not only increases planting costs but also leads to increasingly serious environmental problems. Chinese rice scientists have recently discovered that rice GROWTH-REGULATING FACTOR 4 (GRF4) is a positive regulator of plant carbon-nitrogen metabolism; it also promotes and integrates plant nitrogen metabolism, photosynthesis, and growth. GRF4 can also interact with the rice growth inhibitor DELLA protein; the accumulation of DELLA protein led to the first Green Revolution. By tilting the balance of GRF4-DELLA to increase the abundance of GRF4, it is possible to increase the nitrogen use efficiency of Green Revolution varieties and increase grain yield, while maintaining the excellent traits of semi-dwarfing. Similarly, researchers recently discovered another key gene, NITROGEN-MEDIATED TILLER GROWTH RESPONSE 5 (NGR5) (Wu et al., 2020), which controls the efficiency of nitrogen use in rice. Researchers have confirmed that NGR5 is a positive regulator of rice growth and development (plant height, tiller, and number of grains per panicle) in response to nitrogen. In the current rice varieties, increasing the expression of NGR5 improves nitrogen use efficiency and maintains the semi-dwarfing and high-yield characteristics, ultimately leading to higher rice yields under appropriately reduced nitrogen fertilizer conditions. The discovery of GFR4 and NGR5 genes is expected to lead to the production of new green and high-yield rice varieties.

## 4 Concluding remarks and future perspectives

As China is the largest country engaged in rice research worldwide, the government has made continuous and stable funds available for rice research (Chen et al., 2022). This article summarizes seven representative research areas based on their impact on food security (**Figure 1**). These studies were stimulated by and promoted the realization of Yuan’s two dreams to some extent. For example, the *de novo* domesticated allotetraploid rice is tall, which is conducive to the realization of the dream, “enjoy the cool underneath the rice crops”; and the sea rice developed by Yuan’s research team has laid the foundation for the realization of “hybrid rice to be grown all over the world.” Yuan’s two dreams essentially correspond to the Sustainable Development Goal 2 (Zero Hunger), which was his dream as a youth.

Yuan and his team also conducted fruitful research on low cadmium-accumulating rice (Tang et al., 2017) and sea rice in recent years, both of which originated from their research on hybrid rice. Yuan’s success inspired Chinese scientists engaged in rice research to continually make breakthroughs, as described in this article (**Figure 1**), and will continue to inspire them into the future. The purpose of this article was not to provide a detailed interpretation of these studies, but to summarize the highlights of these studies from the perspective of food security and sustainability in a holistic manner (Gao, 2021). At present, hundreds of millions of people in the world still face hunger every year; this number reached 768 million in 2020, an increase of 118 million from 2019 (FAO et al., 2021). Especially in the context of the raging COVID-19 pandemic, climate change, loss of biodiversity, population surge, and continuing reduction of arable land (Gao et al., 2020), developing countries and low-income populations face greater risks of hunger and malnutrition. Promoting research on the biotechnology of diverse crops, including rice, wheat, corn, and soybeans (Altman et al., 2021; Varshney et al., 2021), is critical to ensuring the world’s food security and achieving the United Nations Sustainable Development Goals (Qaim, 2020).

## Data availability statement

The original contributions presented in the study are included in the article/supplementary material. Further inquiries can be directed to the corresponding author.

## Author contributions

J-GG wrote the paper, and X-GZ revised the paper. Both authors contributed to the article and approved the submitted version.
